# A high efficiency silicon nitride waveguide grating coupler with a multilayer bottom reflector

**DOI:** 10.1038/s41598-019-49324-5

**Published:** 2019-09-10

**Authors:** Jianxun Hong, Andrew M. Spring, Feng Qiu, Shiyoshi Yokoyama

**Affiliations:** 1 Institute for Materials Chemistry and Engineering, Kyushu University, 6-1 Kasuga-koen Kasuga-city, Fukuoka 816-8580 Japan; 2 Department of Molecular and Material Sciences, Kyushu University, 6-1 Kasuga-koen Kasuga-city, Fukuoka 816-8580 Japan; 3 School of Information Engineering, Wuhan University of Technology, Wuhan, 430070 China

**Keywords:** Nanophotonics and plasmonics, Optoelectronic devices and components, Silicon photonics

## Abstract

We propose a high efficiency apodized grating coupler with a bottom reflector for silicon nitride photonic integrated circuits. The reflector consists of a stack of alternate silicon nitride and silicon dioxide quarter-wave films. The design, fabrication and optical characterization of the couplers has been presented. The measured fiber to detector insertion loss was −3.5 dB which corresponds to a peak coupling efficiency of −1.75 dB. A 3 dB wavelength bandwidth of 76.34 nm was demonstrated for the grating coupler with a 20-layer reflector. The fabrication process is CMOS-compatible and requires only a single etching step.

## Introduction

Silicon nitride (SiN_x_) has recently attracted increasing attention and has emerged as an alternative material for photonic integrated circuits (PICs)^1^. Although it has a refractive index of around 1.7–2.3 smaller than that of silicon, it is sufficient for compact integration and high optical power confinement. The moderately small index contrast can reduce the scattering loss due to the sidewall roughness, and allows a high fabrication tolerance^2,3^. The SiN_x_ is transparent through both visible and infrared spectra^4^, and can be readily prepared by conventional deposition technologies^5^.

In order to obtain wafer-scale testing flexibility and facile coupling operation, waveguide grating couplers have been used in Si and SiN_x_ PICs^6–12^. Compared to a silicon grating coupler, SiN_x_ grating couplers have a lower coupling efficiency due to a lower index contrast^2,9,13^. In order to improve the coupling efficiency, two problems must be addressed. One of the problems is that a majority of the optical power is reflected downward into the substrate and dissipates. Bottom reflectors such as a metal mirror^12,14^ and distributed Bragg reflector^3,9,15^ have been utilized to improve the coupling efficiency of silicon and silicon nitride grating couplers. Linear silicon grating reflectors have also been adopted to achieve a higher directionality^2,16^. However, this involves several pattern steps and multiple waveguide layers. Another problem is that the field distribution of the coupled out beam from a uniform SiN_x_ grating coupler does not match the field distribution of a single mode fiber (SMF)^2,11^. This mismatch limits the coupling efficiency. Apodizing of the grating can facilitate a matching of the field distributions.

In this study, we have introduced a bottom multilayer reflector and period apodization to improve the coupling efficiency of the SiN_x_ grating couplers. The reflector consists of a stack of alternate SiN_x_ and SiO_2_ quarter-wave layers, which can be deposited by using the liquid source chemical vapor deposition (LSCVD) technique. The main advantages of the proposed waveguide grating coupler are a high coupling efficiency, versatile design, simple fabrication and complementary metal-oxide semiconductor (CMOS) compatibility.

## Results and Discussion

**Figure 1:**
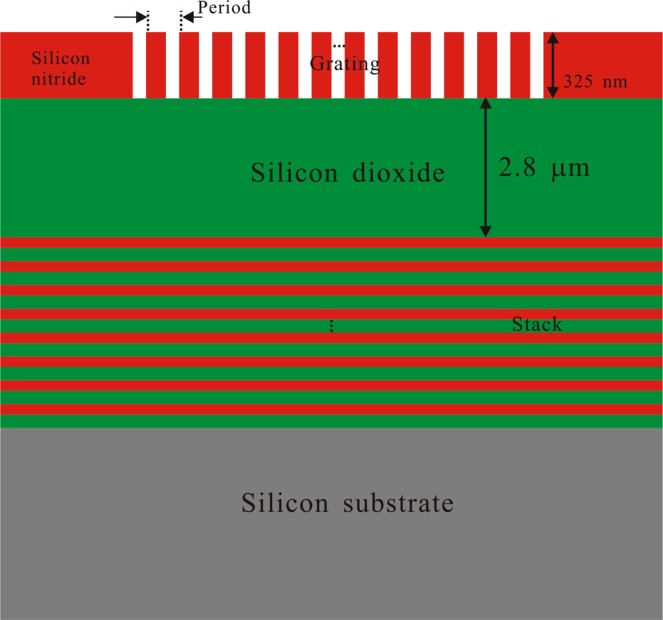
Schematic illustration of the grating coupler with a bottom reflector. The multilayer reflector consists of alternate SiO_2_ and SiN_x_ layers.

Figure 1 shows the waveguide grating coupler with a bottom multilayer reflector on a silicon nitride platform. A stack of the multilayer is deposited on the Si substrate and cladded by a buried oxide (BOX) buffer layer. It consists of a SiN_x_ layer at the buffer layer side, followed by alternate SiO_2_ and SiN_x_ layers. Refractive indices of SiO_2_, SiN_x_ and Si are 1.45, 1.80 and 3.45, respectively. The stack of SiO_2_ and SiN_x_ layers can be fabricated by manipulating the recipe of the LSVCD machine at the same temperature. As a consequence of this deposition technique, the multilayer can be easily fabricated. Furthermore, the low index contrast between SiO_2_ and SiN_x_ provides a good fabrication tolerance for film thickness control. The thickness of the top silicon nitride waveguide layer is 325 nm.

### Multilayer reflector design

**Figure 2:**
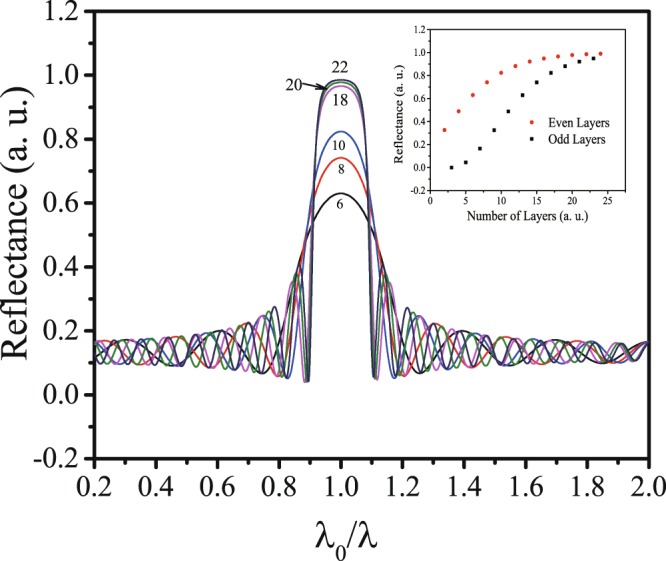
Reflection spectra of quarter-wave multilayer stacks. The number of layers is shown as a parameter on the curves. The insert is the reflectance at the center wavelength for the multilayer stacks with different layers.

The bottom reflector consists of a stack of a multilayer film with alternate SiO_2_ and SiN_x_ layers. This type of multilayer film can be modeled by the transfer matrix method^17^. For the theoretical discussion, we denote the wavelength and center wavelength by *λ* and *λ*_0_, respectively. While all layers in the stack are one-quarter of a wavelength (*λ*_0_) thick, the multilayer system performs as a highly reflective optical film. The reflectance of the quarter-wave stack at *λ*_0_ can be simply expressed as follows.

1 $$R={[\frac{{n}_{{\rm{clad}}}-{(\frac{{n}_{\mathrm{SiN}}}{{n}_{{{\rm{SiO}}}_{2}}})}^{2p}(\frac{{n}_{\mathrm{SiN}}^{2}}{{n}_{{\rm{sub}}}})}{{n}_{{\rm{clad}}}+{(\frac{{n}_{\mathrm{SiN}}}{{n}_{{{\rm{SiO}}}_{2}}})}^{2p}(\frac{{n}_{\mathrm{SiN}}^{2}}{{n}_{{\rm{sub}}}})}]}^{2}\,{\rm{for}}\,N=2p+1,$$

2 $$R={[\frac{{n}_{{\rm{clad}}}-{(\frac{{n}_{\mathrm{SiN}}}{{n}_{{{\rm{SiO}}}_{2}}})}^{2p}{n}_{{\rm{sub}}}}{{n}_{{\rm{clad}}}+{(\frac{{n}_{\mathrm{SiN}}}{{n}_{{{\rm{SiO}}}_{2}}})}^{2p}{n}_{{\rm{sub}}}}]}^{2}\,{\rm{for}}\,N=2p,$$

where *N* is the number of layers, *p* is an integer, *n*_SiO2_, *n*_SiN_, *n*_clad_ and *n*_sub_ are the refractive indices of silicon dioxide, silicon nitride, the cladding and substrate, respectively. Figure 2 shows the calculated reflection spectra of quarter-wave stacks by using the transfer matrix method. The plateau centered at *λ*_0_ offers a high-reflectance zone within which the reflectance at each wavelength increases monotonically with increasing *N*, and approaching 1.0 as *N* tends to infinity. The width of the high-reflectance zone is near 0.2*λ*_0_. The multilayer effectively functions as a broadband reflector within this zone. The insert of Fig. 2 shows the reflectance of quarter-wave stacks with different layers at *λ*_0_ calculated by using Eqs (1) and (2). It can be observed that the films with an even number of layers have higher reflectance than the films with an adjacent odd number of layers. So, we selected *N* to be 18 or 20 for the fabrication.

### Grating optimization and apodization design

Firstly, the period and the BOX thickness of the uniform silicon nitride grating coupler were optimized^8^. Subsequently, the apodization was designed near the optimized values. Here, we analyze and discuss the coupling efficiency from grating to single mode fiber by assuming the coupling efficiency from fiber to grating is the same. The thicknesses of the alternate SiO_2_ and SiN_x_ layers are set to be 258.6 nm and 208.3 nm, which are exactly a quarter-wave thickness at *λ*_0_ = 1.55 μm. For the first order Bragg diffraction, the grating period can be simplified as^3^

3 $$\Lambda =\frac{{\lambda }_{0}}{{n}_{{\rm{eff}}}-{n}_{{\rm{c}}}\,\sin \,\theta }$$

where, *n*_c_ is the refractive index of the cladding material, *θ* is the coupling angle, *n*_eff_ is the effective refractive index and can be estimated by^18^

4 $${n}_{{\rm{eff}}}=ff{n}_{{\rm{eff}}1}+(1-ff){n}_{{\rm{eff}}2}$$

where *ff* is the fill factor defined as the ratio of the grating teeth to the period, *n*_eff1_ and *n*_eff2_ are the effective refractive index of the grating teeth and the grating slots, respectively.

**Figure 3:**
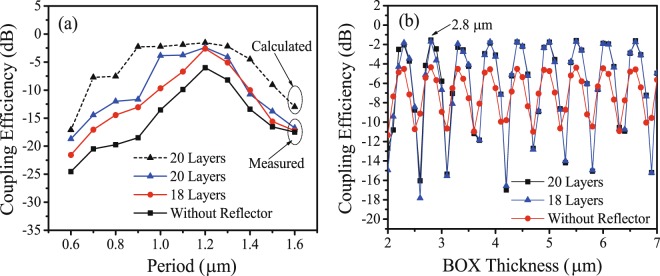
Coupling efficiencies of the grating couplers. (**a**) Calculated (dashed line) and measured (solid lines) coupling efficiencies as a function of the grating period. (**b**) Calculated coupling efficiencies as a function of the thickness of the BOX layer.

In order to compare the calculation with experiment results for period optimization, we also fabricated grating couplers with periods from 0.6 μm to 1.5 μm. The coupling efficiencies of couplers with different periods are shown in Fig. 3(a). It can be observed that the coupling efficiency is highly dependent on the grating period. The maximum coupling efficiencies were obtained at the period of 1.2 μm in both calculation and experiment for a standard uniform grating coupler.

The thickness of the BOX layer also has an important impaction on the coupling efficiency. Figure 3(b) shows the dependence of the coupling efficiency on the BOX thickness for grating couplers with different layers in the reflector. It can be observed that the patterns for different values of *N* are similar. BOX thicknesses of the peaks for different *N* are also similar. Therefore, we selected the BOX thickness as 2.8 μm. Because of the oscillation in the pattern, the thickness must be controlled precisely.

**Figure 4:**
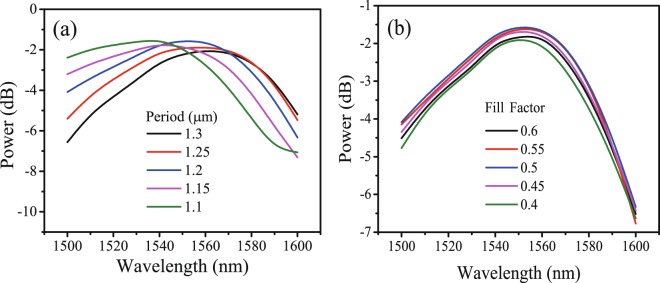
Calculated spectra of grating couplers with *N* = 20. (**a**) Spectra of grating couplers with different periods. The fill factor of the grating is set as 0.5. (**b**) Spectra of grating couplers with different fill factors. The period of the grating is set as 1.2 μm.

The spectra of grating couplers with different periods and fill factors were calculated and are shown in Fig. 4. It can be observed that the spectrum shifts to a longer wavelength with the period and fill factor increasing. According to Eqs (3) and (4), the effective refractive index increases with the fill factor, which results in a spectrum shifting. The peak coupling efficiency also changes with the period and fill factor. Moreover, the period has a much larger impact on the spectrum and coupling efficiency than the fill factor. In order to maximize the coupling efficiency at a specific wavelength, both the period and fill factor must be optimized. Here, the period and fill factor were selected as 1.2 μm and 0.5, respectively. In this case, the 3 dB and 1 dB bandwidths are 82 nm and 54 nm, respectively.

**Figure 5:**
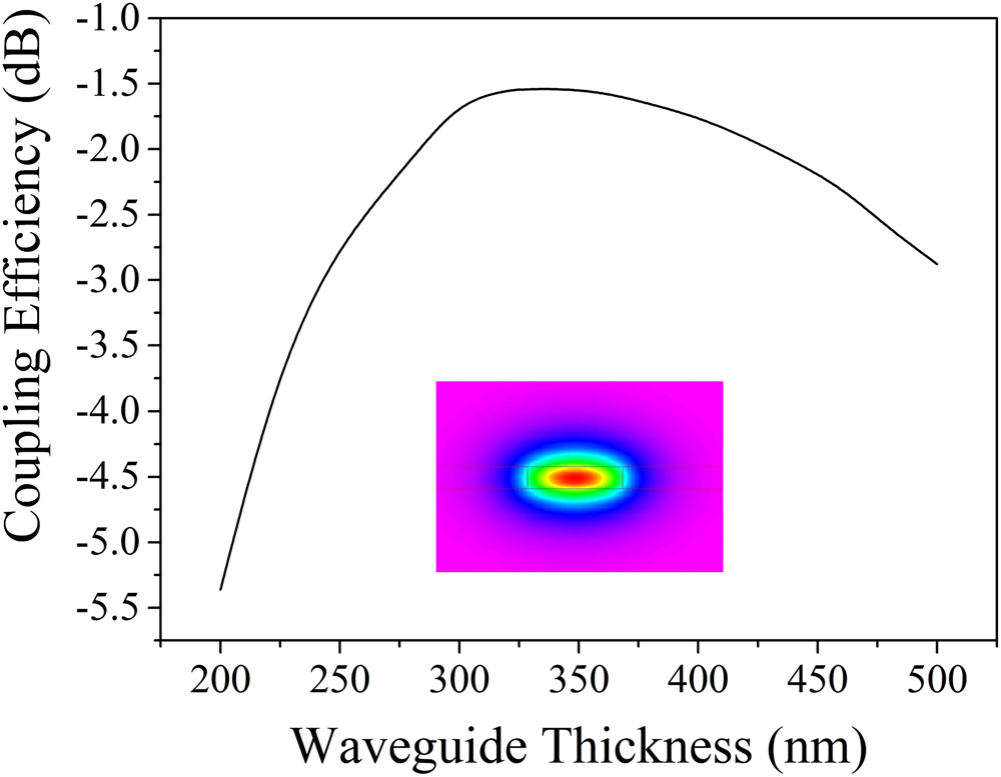
Calculated coupling efficiencies of grating couplers with a different waveguide thickness. The period, fill factor and *N* are 1.2 μm, 0.5 and 20, respectively.

The coupling efficiency dependence on the thickness of the top silicon nitride layer was calculated and is shown in Fig. 5. It can be observed that the thickness of 325 nm is near the center of the maximum region. Therefore, the thickness of the top silicon nitride was selected as 325 nm in this investigation. The silicon nitride waveguide with a width of 1.0 μm and a thickness of 325 nm functions in a single mode condition. The insert of Fig. 5 shows the field distribution of the fundamental mode.

The coupling efficiency can also be improved further by apodizing the period and the fill factor of the grating^11^. The optical power guided in the uniform grating structure can be described as^19,20^

5 $$P={P}_{0}{e}^{-2\alpha z}$$

where, *α* is the field leakage factor, *P*_0_ is power of the guided wave at the beginning of the grating. *dP* is the power due to the harmonic fields radiate away from the waveguide. Therefore, the radiated out power can be expressed as^19,20^

6 $${P}_{{\rm{rad}}}=-\,\frac{dP}{dz}=2\alpha {P}_{0}{e}^{-2\alpha z}$$

Note, *P*_rad_ is the power per unit length along *z* direction. The leakage factor *α* can be extracted from the radiation power density data by using Eq. (6).

In order to obtain a Gaussian output electric field profile *G* (*z*), the leakage factor *α* is varied along the *z* direction to make *P*_rad_ = *G*^2^(*z*)^20–22^. The dependence of the leakage factor 2*α* on the grating length can be expressed as

7 $$2\alpha =\frac{{G}^{2}(z)}{{P}_{0}-{\int }_{0}^{z}\,{G}^{2}(z)dz}$$

where, *G* (*z*) is used to describe the Gaussian field profile of a standard single mode fiber with a mode field diameter (MFD) of 10.4 μm. Neglecting the radiation into the substrate, ${\int }_{0}^{{z}_{1}}\,{G}^{2}(z)dz=\eta {P}_{0}$. Here, *η* is the coupling efficiency, the beginning and the end of the grating are assumed at 0 and *z*_1_, respectively.

**Figure 6:**
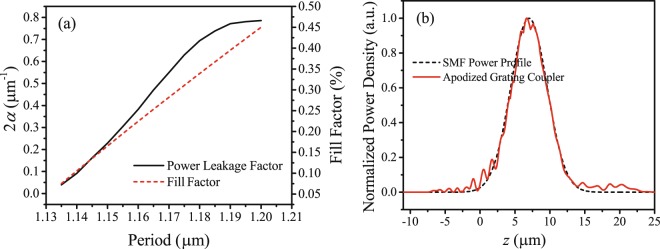
The apodization results of the grating couplers. (**a**) Calculated power leakage factor and the required fill factor of a grating cell as a function of grating period. (**b**) The coupling out field profile of the apodized grating and the Gaussian fiber field profile with an MFD of 10.4 μm.

The leakage factor *α* for gratings with different periods were calculated by using 2D simulation and Eq. (6). The result is shown in Fig. 6(a). During simulation, the fill factor was determined by carefully checking the scattering direction for every period. In this case, the Bloch condition is satisfied, which can ensure the phase match between adjacent grating cells in the apodized grating coupler.

Then, the required period and fill factor distribution for the apodized grating coupler were determined according to Eq. (7) and Fig. 6(a). The periods and fill factors (in brackets) from the front to the back end are 1.135 μm (0.086 μm), 1.14 μm (0.12 μm), 1.147 μm (0.16 μm), 1.153 μm (0.212 μm), 1.161 μm (0.26 μm), 1.168 μm (0.316 μm), 1.175 μm (0.365 μm), 1.182 μm (0.413 μm), 1.191 μm (0.47 μm), 1.2 μm (0.54 μm), 1.191 μm (0.47 μm) and 1.182 μm (0.413 μm), followed by a 9-period uniform grating of 1.2 μm (0.54 μm). The output field distribution profile of the designed apodized grating coupler is shown in Fig. 6(b). It overlaps well with the SMF Gaussian profile which ensures a high coupling efficiency.

**Figure 7:**
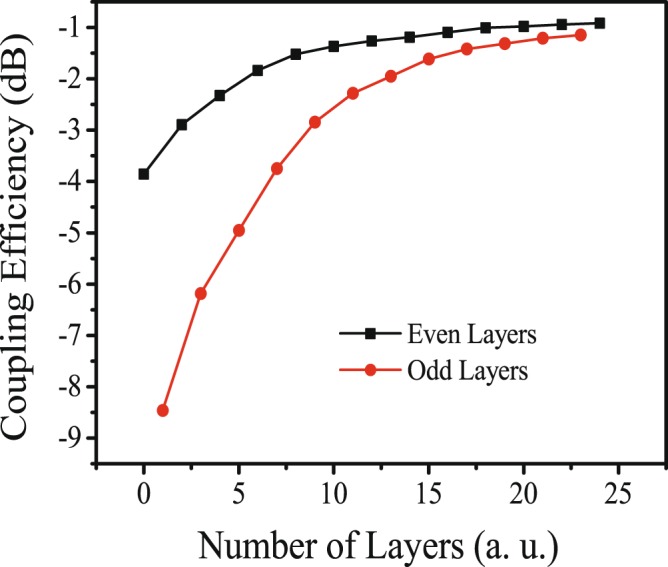
Calculated coupling efficiencies of grating couplers with different bottom reflector layers. Grating apodization and optimized BOX thickness were adopted in this calculation.

After the grating optimization and bottom reflector design, coupling efficiencies of grating couplers with different *N* were calculated as shown in Fig. 7. As can be observed, couplers with an even number of bottom layers showed a higher coupling efficiency than the couplers with an adjacent odd number of bottom layers. The tendency agreed well with that shown in Fig. 2. The coupling efficiency increased up to −1.0 dB with increasing *N*, and reached a plateau for *N* > 12.

### Fabrication and coupling efficiency measurement

**Figure 8:**
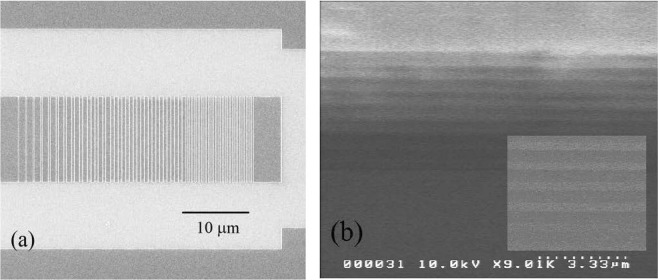
SEM pictures of the device. (**a**) SEM top view of the grating coupler. (**b**) SEM cross-section of the stack of 18 layers. The insert shows the zoom-in image including 10 layers. The bright and dark strips are SiN_x_ and SiO_2_ layers, respectively.

The scanning electron microscopy (SEM) images of the fabricated waveguide are shown in Fig. 8. A 20-period grating with a short period of 0.52 μm was arranged at the end of the coupler to reflect back the escaping power. The periods and fill factor of the grating fit the designed pattern well. The input and output grating couplers were linked by a standard 3.0 mm long strip waveguide with a width of 1.0 μm. The coupler had a 13 μm-wide and 30 μm-long structure. The mode in the strip was transformed adiabatically to the grating coupler waveguides via a 500 μm-long taper.

We characterized the coupling efficiencies of grating couplers by measuring the laser to detector insertion losses. The grating couplers diffract light into/from cleaved single mode fibers tilted at an angle of 8° with respect to the grating surface normal. Usually, the transmission loss of the silicon nitride strip should be subtracted from the insertion loss^2,22–24^. The coupling efficiency is half of the insertion loss after subtraction. The propagating loss was measured to be 2.4 dB/cm by comparing straight waveguides with different lengths. The measured coupling efficiencies at 1550 nm of apodized and unapodized couplers with different bottom reflectors are summarized in Table 1. The coupling efficiency increases with *N* as expected from the calculation. The coupling efficiency of the grating couplers with 18 and 20 layers are very close which agrees well with the simulation. By comparing with the standard grating coupler, the coupling efficiency was improved by 3.65 dB. It can be observed that the bottom reflector can improve the coupling efficiency by approximately 3.2 dB and the apodization can improve the coupling efficiency by approximately 0.7 dB.

**Table 1:** Measured coupling efficiencies at a wavelength of 1550 nm.

Number of layers (*N*)	Coupling efficiency (dB)	
	Apodized	Unapodized
0	−4.9	−5.6
18	−2.07	−2.6
20	−1.95	−2.4

**Figure 9:**
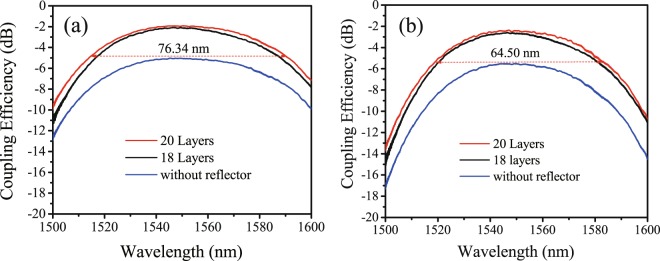
Measured transmission spectra. (**a**) Spectra of the apodized grating couplers with reflectors; (**b**) Spectra of the unapodized grating couplers with reflectors.

Figure 9(a) shows the transmission spectra of the apodized grating couplers (*N* = 0, 18 and 20). The light from a tunable laser (SANTEC TSL550) was coupled into the grating coupler. The wavelength was swept over a range of 100 nm and the spectrum recorded by an optical power meter (SANTEC MPM200) with 1 pm wavelength resolution. The measured bandwidth of grating couplers with reflectors is almost identical to that of the standard grating coupler without a reflector. The 3 dB bandwidth of the grating coupler with *N* = 20 is 76.34 nm, which covers most of operation frequencies of C-band and L-band. The corresponding 1 dB bandwidth is 52.5 nm, which is smaller than the reported 80 nm of the Si_3_N_4_-on-SOI dual-level grating coupler^16^. We attributed the smaller 1 dB bandwidth to the non-flat high-reflectance zone of the bottom multilayer reflector as shown in Fig. 2. Even though the width of the high-reflectance zone is as large as several hundred nanometers, the reflectance characteristic is not totally flat. For comparison, the transmission spectra of the unapodized grating couplers are also shown in Fig. 9(b). It can be observed that the apodization can slightly broaden the bandwidth of the grating couplers because more periods were used in apodized grating couplers^18^.

It should be noted that no index matching fluid was applied between the gratings and fibers during the measurements for practical convenience and repeated use. However, an estimate of the excess loss due to the use of air coupling would be beneficial in understanding the performance of the device. It was evaluated experimentally that the coupling efficiency can be improved by about 0.35 dB while the index matching fluid was used as the waveguide top cladding and filled the gap between the gratings and fibers^25^. In our experimental samples, SiO_2_ films were deposited as a top cladding. In order to evaluate the improvement effect of the index matching fluid, we dropped the index matching fluid on the surface to fill the gaps between the gratings and fibers and measured the coupling efficiency. Results show that the coupling efficiency was improved by about 0.20 dB. Taking into account this improvement, the peak coupling efficiency of the proposed grating coupler is −1.75 dB.

## Conclusions

An efficient apodized SiN_x_ waveguide grating coupler with a bottom multilayer reflector was theoretically and experimentally demonstrated. Apodization and a multilayer reflector were adopted to improve the coupling efficiency. The reflector was designed by using alternate quarter-wave SiN_x_ and SiO_2_ layers. The waveguide was fabricated by one EBL and ICP etching step. Results show that the bottom reflector and the grating apodization were able to enhance the coupling efficiency by approximately 3.2 dB and 0.7 dB, respectively. A high coupling efficiency of −1.75 dB with 3 dB bandwidth of 76.34 nm has been demonstrated. This approach facilitates the design and fabrication process of the grating couplers with a bottom reflector.

## Methods

This multilayer bottom reflector was modeled by the general transfer matrix method. The reflectance and spectra were calculated by using C-language programing. For design and optimization of the grating coupler with a bottom multilayer reflector, we used the two-dimensional (2D) finite difference time domain method (FDTD) by using the commercial software Rsoft^8,11,12,26^.

In the fabrication, a silicon wafer was used as the substrate. The SiN_x_ and SiO_2_ films were deposited using the LSCVD (SAMCO) at 150 °C. LSCVD can deposit silicon nitride at low temperature by using N_2_ instead of NH_3_ as the deposition precursor, which decreases the concentration of the dangling hydrogen bonds and the tensile stress in the film^27,28^. There is no obvious absorption peak near 1520 nm which exists in silicon nitride films deposited by plasma enhanced chemical vapor deposition (PECVD)^29^. Moreover, no cracks were observed over the entire sample. We prepared bottom reflectors with *N* = 0, 18 and 20 for comparison. The waveguide and grating structures were patterned by electronic beam lithography (EBL) and ion coupling plasma (ICP) with CHF_3_ gas.
